# Triglyceride Levels Are Closely Associated with Mild Declines in Estimated Glomerular Filtration Rates in Middle-Aged and Elderly Chinese with Normal Serum Lipid Levels

**DOI:** 10.1371/journal.pone.0106778

**Published:** 2014-10-02

**Authors:** Xinguo Hou, Chuan Wang, Xiuping Zhang, Xiangmin Zhao, Yulian Wang, Chengqiao Li, Mei Li, Shaoyuan Wang, Weifang Yang, Zeqiang Ma, Aixia Ma, Huizhen Zheng, Jiahui Wu, Yu Sun, Jun Song, Peng Lin, Kai Liang, Lei Gong, Meijian Wang, Fuqiang Liu, Wenjuan Li, Juan Xiao, Fei Yan, Junpeng Yang, Lingshu Wang, Meng Tian, Jidong Liu, Ruxing Zhao, Shihong Chen, Li Chen

**Affiliations:** 1 Department of Endocrinology of Qilu Hospital and Institute of Endocrinology and Metabolism, Shandong University, Jinan, Shandong, China; 2 Shantui Community Health Center, Jining, Shandong, China; 3 Department of Endocrinology, Second People's Hospital of Jining, Jining, Shandong, China; 4 Lukang Hospital of Jining, Jining, Shandong, China; 5 China National Heavy Duty Truck Group Corporation Hospital, Jinan, Shandong, China; 6 Department of Endocrinology, the Second Hospital of Shandong University, Jinan, Shandong, China; Northeast Ohio Medical University, United States of America

## Abstract

**Objective:**

To investigate the relationship between lipid profiles [including total cholesterol (TC), triglyceride (TG), low-density lipoprotein cholesterol (LDL-C) and high-density lipoprotein cholesterol (HDL-C)] and a mild decline in the estimated glomerular filtration rate (eGFR) in subjects with normal serum lipid levels.

**Design and Methods:**

In this study, we included 2647 participants who were ≥40 years old and had normal serum lipid levels. The Chronic Kidney Disease Epidemiology Collaboration (CKD-EPI) equation was used to estimate the GFR. A mildly reduced eGFR was defined as 60–90 mL/min/1.73 m^2^. First, multiple linear regression analysis was used to estimate the association of lipid profiles with the eGFR. Then, the levels of each lipid component were divided into four groups, using the 25th, 50th and 75th percentiles as cut-off points. Finally, multiple logistic regression analysis was used to investigate the association of different lipid components with the risk of mildly reduced eGFR.

**Results:**

In the group with a mildly reduced eGFR, TG and LDL-C levels were significantly increased, but HDL-C levels were significantly decreased. After adjusting for age, gender, body mass index (BMI), systolic blood pressure (SBP), glycated hemoglobin (HbA_1c_), smoking and drinking, only TC and TG were independently related to the eGFR. Additionally, only TG showed a linear relationship with an increased risk of a mildly reduced eGFR, with the highest quartile group (TG: 108–150 mg/dl [1.22–1.70 mmol/L]) having a significantly increased risk after adjusting for the above factors.

**Conclusions:**

Triglyceride levels are closely associated with a mildly reduced eGFR in subjects with normal serum lipid levels. Dyslipidemia with lower TG levels could be used as new diagnostic criteria for subjects with mildly reduced renal function.

## Introduction

Chronic kidney disease (CKD), characterized by albuminuria or reduced kidney function, is a worldwide public health problem [1], [2] that increases the risk of cardiovascular events and mortality [3], [4]. Even mild renal insufficiency increases the risk of cardiovascular events [5], [6] and is predictive of the progression of kidney disease [7]. Therefore, screening for risk factors is critical during the early stage of CKD, which is characterized by a mildly reduced eGFR.

Dyslipidemia is often detected in patients with CKD and has been shown to mediate atherosclerotic disease. Therefore, statin treatment has been recommended for patients with CKD according to the lipid management guidelines developed by Kidney Disease: Improving Global Outcomes (KDIGO) [8]. However, the specific target range of serum lipid levels remains to be determined. Additionally, most previous studies of the relationship between serum lipid levels and CKD have focused on dyslipidemia and severe kidney disease where the eGFR was less than 60 mL/min/1.73 m^2^ [9]. Few studies have been conducted to determine the association of normal serum lipid levels, as defined by current criteria, with a mildly reduced eGFR. Whether the current criteria for normal serum lipid levels are appropriate for patients with mild renal insufficiency remains to be clarified. Here, to explain this issue, we explored the relationship between lipid profiles and a mildly reduced eGFR in subjects with normal serum lipid levels.

## Materials and Methods

### Ethics Statement

This cross-sectional study is part of the REACTION study [10], [11] and was approved by the Ruijin Hospital Ethics Committee of the Shanghai Jiao Tong University School of Medicine. Written informed consent was obtained from all participants in this study.

### Study population

The present study recruited 10,028 subjects who were ≥40 years old in Shandong province from January to April 2012. We excluded subjects with (1) missing data for calculation of the eGFR; (2) an eGFR <60 mL/min/1.73 m^2^; (3) dyslipidemia (see below); (4) previously diagnosed kidney disease, including autoimmune or drug-induced kidney disease, nephritis, renal fibrosis or renal failure, or subjects who had a kidney transplant and were receiving dialysis treatment; (5) previously diagnosed hepatic disease, including fatty liver, liver cirrhosis and autoimmune hepatitis; and (6) any malignant disease. Finally, 2647 subjects (1734 women) were eligible for the analysis.

### Data collection

Demographic characteristics, lifestyle information and previous medical history were obtained by trained investigators through a standard questionnaire. BMI was calculated as weight (kg) divided by height squared (m^2^). Blood pressure (BP) was measured 3 times consecutively (OMRON Model HEM-752 FUZZY, Omron Company, Dalian, China), and the average reading was used for analysis. After an overnight fasting, venous blood samples were collected between 07:00 and 09:00 for measurement of fasting blood glucose (FBG), creatinine and lipid profiles (TC, TG, LDL-C and HDL-C). Postprandial blood glucose (PBG) was measured after subjects had completed a 75-g OGTT. HbA_1c_ was measured by high-performance liquid chromatography (VARIANT II and D-10 Systems, BIO-RAD, USA).

The estimated GFR (eGFR) was calculated from creatinine levels using the CKD-EPI formula [12].

### Definition

Normal eGFR was defined as ≥90 mL/min/1.73 m^2^; mildly reduced eGFR was defined as 60–90 mL/min/1.73 m^2^.

Diabetes was defined by the 1999 World Health Organization (WHO) criteria [13]: FBG ≥126 mg/dl (7.0 mmol/L) and/or PBG ≥200 mg/dl (11.1 mmol/L) or a history of diabetes.

Dyslipidemia was defined by the 2007 Guidelines for Prevention and Treatment of Dyslipidemia in Adults in China [14]: (1) TC ≥200 mg/dl (5.18 mmol/L); (2) TG ≥150 mg/dl (1.70 mmol/L); (3) LDL-C ≥130 mg/dl (3.37 mmol/L); (4) HDL-C <40 mg/dl (1.04 mmol/L); or (5) the patient was undergoing treatment for any of these conditions.

### Statistical analysis

The continuous variables in this study, which contained a large cohort of patients, exhibited normal distribution or approximately normal distribution and are presented as the means ± SD, and the categorical variables are presented as numbers (%). Differences between groups were analyzed using Student's *t* test for continuous data and the chi-square test for categorical data. After verifying the assumption of a linear relationship between the dependent and independent variables that were introduced into the linear regression model (assessed using a histogram of the residuals, together with a scatter plot of the standardized residuals to the standardized predicted values in different models, as described below), multiple linear regression analysis was used to estimate the association of lipid profiles with the eGFR. Three models were constructed for each component of lipid profiles: the first model was not adjusted; the second model was adjusted for age and gender; and the third model was adjusted for age, gender, BMI, systolic blood pressure (SBP), HbA_1c_, smoking and drinking. The levels of each lipid component were divided into four groups, using the 25th, 50th and 75th percentiles as cut-off points: 163.10, 177.02 and 189.00 mg/dl (4.22, 4.58 and 4.89 mmol/L) for TC; 65.49, 84.08 and 108.00 mg/dl (0.74, 0.95 and 1.22 mmol/L) for TG; 85.97, 98.30 and 109.87 mg/dl (2.23, 2.55 and 2.85 mmol/L) for LDL-C; and 50.44, 57.37 and 65.84 mg/dl (1.31, 1.49 and 1.71 mmol/L) for HDL-C. Then, the associations of the different lipid components (we introduced the ordinal independent variables as quartiles of TC, TG, LDL-C and HDL-C as dummy variables) with the risk of a mildly reduced eGFR were estimated using multiple logistic regression analysis in the same three models mentioned above. *P*-values for the trends were calculated by Spearman correlation analysis of categorical variables and odds ratios for the different groups, scored 0, 1, 2 and 3, respectively. *P*<0.05 was considered statistically significant. All statistical analyses were performed using SPSS 16.0 (SPSS Inc., Chicago, IL, USA).

## Results

### Characteristics of study participants

We included 2647 subjects (1734 women) who were divided into two groups according to the eGFR, using 90 mL/min/1.73 m^2^ as the cut-off value. As shown in Table 1, almost all characteristics were different between the two groups, except for TC. In the group with a mildly reduced eGFR, age, BMI, SBP, DBP, FBG, PBG, HbA_1c_, TG, LDL-C, the percentages of males and diabetics and smoking and drinking statuses among the patients were significantly increased, while the HDL-C level was significantly decreased (all *P*<0.001).

**Table 1:** Characteristics of the study participants.

	eGFR (mL/min/1.73 m^2^)		
Characteristics	≥90 (n = 1665)	(60–90) (n = 982)	*P*-value
Female (%)	77.60%	45.01%	<0.001
Age (years)	51.09±7.73	63.08±9.38	<0.001
BMI (kg/m^2^)	25.26±3.52	26.00±3.48	<0.001
SBP (mmHg)	131.60±19.31	141.92±20.49	<0.001
DBP (mmHg)	77.55±10.93	80.29±11.40	<0.001
FBG (mg/dl)	99.72±22.22	111.49±32.14	<0.001
PBG (mg/dl)	113.05±53.69	136.03±71.38	<0.001
HbA_1c_ (%)	5.81±0.84	6.20±1.21	<0.001
Diabetes (%)	6.79%	18.13%	<0.001
TC (mg/dl)	173.87±17.92	175.27±14.85	0.051
TG (mg/dl)	85.22±27.65	91.97±26.94	<0.001
LDL-C (mg/dl)	95.21±17.46	99.15±17.68	<0.001
HDL-C (mg/dl)	60.21±11.05	56.21±10.53	<0.001
Smoking (%)	8.89%	23.73%	<0.001
Drinking (%)	14.23%	35.13%	<0.001
eGFR (mL/min/1.73 m^2^)	102.29±6.69	78.47±7.86	<0.001

Data are the means ± SD or numbers (%). BMI, body mass index; SBP, systolic blood pressure; DBP, diastolic blood pressure; FBG, fasting blood glucose; PBG, postprandial blood glucose; TC, total cholesterol; TG, triglyceride; LDL-C, low-density lipoprotein cholesterol; HDL-C, high-density lipoprotein cholesterol; eGFR, estimated glomerular filtration rate.

### Multiple linear regression analysis

As shown in Table 2, we constructed three models to analyze the association of each lipid component with the eGFR (the 4 lipid components were analyzed separately due to colinearity). The assumption of a linear relationship between each lipid component and the eGFR was assessed using a histogram of the residuals, together with a scatter plot of the standardized residuals to the standardized predicted value in the different models, showing an approximately linear relationship, especially in models 2 and 3. In model 1, the TC, TG, LDL-C and HDL-C levels were independently related to the eGFR. However, after adjusting for age and gender, HDL-C levels were no longer related to the eGFR in model 2. Finally, in model 3, only TC and TG were chosen as independent variables in subjects with normal serum lipid levels.

**Table 2:** The association of lipid profiles with the eGFR.

Models	Independent variable	β Coefficient	95% CI	*P*-value
Model 1				
Model 1a	TC, mg/dl	−0.061	−0.090 to −0.033	**<0.001**
Model 1b	TG, mg/dl	−0.074	−0.093 to −0.056	**<0.001**
Model 1c	LDL-C, mg/dl	−0.105	−0.134 to −0.076	**<0.001**
Model 1d	HDL-C, mg/dl	0.227	0.181 to 0.273	**<0.001**
Model 2				
Model 2a	TC, mg/dl	−0.029	−0.045 to −0.014	**<0.001**
Model 2b	TG, mg/dl	−0.024	−0.034 to −0.014	**<0.001**
Model 2c	LDL-C, mg/dl	−0.025	−0.040 to −0.009	**0.002**
Model 2d	HDL-C, mg/dl	0.017	−0.008 to 0.043	0.179
Model 3				
Model 3a	TC, mg/dl	−0.028	−0.044 to −0.013	**<0.001**
Model 3b	TG, mg/dl	−0.017	−0.027 to −0.006	**<0.001**
Model 3c	LDL-C, mg/dl	−0.012	−0.028 to 0.003	0.125
Model 3d	HDL-C, mg/dl	−0.012	−0.038 to 0.014	0.356

Model 1: not adjusted.

Model 2: adjusted for age and gender.

Model 3: adjusted for age, gender, BMI, SBP, HbA_1c_, smoking and drinking.

### Multiple logistic regression analysis

As shown in Table 3, we analyzed the association of increased lipid profiles with the risk of a mildly reduced eGFR in three models. In model 1, both TG and LDL-C levels were positively related, but HDL-C levels were negatively related, to an increased risk of a mildly reduced eGFR. In contrast, there was no linear relationship between TC levels and an increased risk of a mildly reduced eGFR, though the highest quartile group of TC levels had a significantly increased risk (*P* = 0.035). However, after adjusting for age and gender (model 2) or further adjusting for BMI, SBP, HbA_1c_, smoking and drinking (model 3), only TG showed a linear relationship with an increased risk of a mildly reduced eGFR, with the highest quartile group (TG: 108–150 mg/dl [1.22–1.70 mmol/L]) significantly increasing the risk.

**Table 3:** The association of lipid profiles with the risk of a mildly reduced eGFR.

	Model 1		Model 2		Model 3	
Independent variable	Odds ratio (95% CI)	*P*-value	Odds ratio (95% CI)	*P*-value	Odds ratio (95% CI)	*P*-value
TC						
Q1	1		1		1	
Q2	1.05 (0.84–1.32)	0.664	0.93 (0.65–1.32)	0.665	0.97 (0.67–1.39)	0.855
Q3	1.00 (0.80–1.25)	0.973	0.90 (0.63–1.28)	0.558	0.91 (0.64–1.30)	0.608
Q4	1.27 (1.02–1.59)	**0.035**	1.21 (0.86–1.70)	0.276	1.23 (0.87–1.74)	0.237
*P* for trend	1.00		0.80		0.80	
TG						
Q1	1		1		1	
Q2	1.41 (1.11–1.78)	**0.004**	1.33 (0.92–1.92)	0.129	1.25 (0.86–1.81)	0.245
Q3	1.82 (1.44–2.30)	**<0.001**	1.43 (1.00–2.06)	0.052	1.31 (0.90–1.91)	0.154
Q4	1.98 (1.57–2.49)	**<0.001**	1.78 (1.25–2.53)	**0.001**	1.61 (1.12–2.32)	**0.011**
*P* for trend	**<0.01**		**<0.01**		**<0.01**	
LDL-C						
Q1	1		1		1	
Q2	1.03 (0.82–1.30)	0.803	0.89 (0.62–1.28)	0.523	0.92 (0.64–1.33)	0.655
Q3	1.47 (1.18–1.84)	**0.001**	1.25 (0.89–1.76)	0.204	1.21 (0.85–1.71)	0.295
Q4	1.76 (1.41–2.20)	**<0.001**	1.29 (0.92–1.81)	0.141	1.19 (0.84–1.68)	0.334
*P* for trend	**<0.01**		0.20		0.20	
HDL-C						
Q1	1		1		1	
Q2	0.69 (0.55–0.85)	**0.001**	0.92 (0.66–1.27)	0.604	0.96 (0.69–1.34)	0.818
Q3	0.47 (0.37–0.59)	**<0.001**	0.81 (0.58–1.13)	0.207	0.85 (0.60–1.20)	0.344
Q4	0.38 (0.31–0.48)	**<0.001**	0.85 (0.60–1.20)	0.341	1.02 (0.71–1.46)	0.936
*P* for trend	**<0.01**		0.20		0.80	

Model 1: not adjusted.

Model 2: adjusted for age and gender.

Model 3: adjusted for age, gender, BMI, SBP, HbA_1c_, smoking and drinking.

## Discussion

A recent study revealed that patients with CKD have cardiovascular mortality rates at least 10 times higher than those of the general population [15] and that dyslipidemia may play an important role in mediating cardiovascular disease and many other complications of CKD [16]. Therefore, an increasing number of experts suggest lipid-lowering therapies in patients with CKD [8], [17], [18]. Additionally, more and more studies have indicated that lipid-lowering therapy clearly affects the incidence of cardiovascular disease, total mortality, stroke, and myocardial infarction in patients with CKD [19], [20]. However, the specific target range of serum lipid levels remains to be determined.

Aside from advanced CKD, a mild reduction of the eGFR was also observed to increase the risk of cardiovascular events, such as arterial stiffness, coronary artery calcium, higher rates of stress-induced ischemia, myocardial hypertrophy, and even mortality [5], [6], [21], [22], [23]. Moreover, two cross-sectional studies performed in China found that the percentage of dyslipidemia was significantly higher in patients with a mildly reduced eGFR than in subjects with a normal eGFR [24], [25], suggesting that dyslipidemia might also be closely associated with a mildly reduced eGFR. Therefore, it is important to identify a specific range of normal serum lipid levels for subjects with a mildly reduced eGFR. However, the association of normal serum lipid levels, as defined by the current criteria, with a mildly reduced eGFR remains unclear; if they are closely associated, new criteria for dyslipidemia might need to be determined.

In the present study, we found that TG and LDL-C levels were significantly increased but HDL-C levels were significantly decreased in the group with a mildly reduced eGFR in middle-aged and elderly Chinese subjects with normal serum lipid levels. The traditional risk factors for CKD include age, gender, overweight, hypertension, diabetes, smoking and drinking [2], [26], [27], [28]. Therefore, we adjusted for age, gender, BMI, SBP, HbA1c, smoking and drinking to analyze the association of lipid profiles with the eGFR and the risk of a mildly reduced eGFR, and we found that TC and TG levels were significantly associated with a decreased eGFR, independently of the above risk factors. Moreover, only TG showed a linear relationship with an increased risk of a mildly reduced eGFR, with the highest quartile group (TG: 108–150 mg/dl [1.22–1.70 mmol/L]), significantly increasing the risk after adjusting for the factors mentioned above. All of the results suggest that even within a normal range of serum lipid levels, as defined by the current criteria, TG significantly increased the risk of a mildly reduced eGFR, indicating that we should pay more attention to controlling TG levels to prevent the progression of CKD. Additionally, new criteria for dyslipidemia might need to be determined in subjects with mildly reduced eGFR.

Creatinine-based equations for estimating the GFR include the Cockcroft-Gault equation proposed in 1976 [29], the Modification of Diet in Renal Disease (MDRD) study equation proposed in 1999 [30] and the CKD-EPI equation proposed in 2009 [11]. Currently, the Cockcroft-Gault equation has been supplanted by the MDRD study equation and the CKD-EPI equation [31]. A recent study performed in South Asians, aged 40 years or older as in the present study, demonstrated that the CKD-EPI equation was more accurate and precise in estimating the GFR than the MDRD study equation [32]. Therefore, we selected the CKD-EPI equation to calculate the eGFR.

Of course, our study has some limitations. First, a cross-sectional study cannot infer causality between lipid profiles and a mildly reduced eGFR, so whether the decline in the eGFR produces the high levels of TG and cholesterol, or the inverse, where the high levels of TG and cholesterol lead to a decline in the eGFR, remains unknown. Second, we could not provide sufficient evidence to change the current dyslipidemia criteria. Though a dose-dependent effect was observed between TG and a reduced eGFR in this study (the higher the TG levels, the higher the risk for a reduced eGFR, Table 3), the causality between lipid profiles and a mildly reduced eGFR remains unclear. Moreover, there was not good evidence that pharmacological treatment effects of high triglycerides on cardiovascular outcomes differed between those with and without lower baseline eGFR[33]. Therefore, more randomized controlled trials are needed to clarify the specific range of normal serum lipid levels for patients with a mildly reduced eGFR at baseline to prevent the progression of CKD and related cardiovascular complications. Third, the risk was not very high for the highest quartile of TG (OR = 1.61), further adjustments for other unknown risk factors may drop the risk to 1, as was observed with TC, LDL-C and HDL-C when adjusted for the risk factors considered in this study. Therefore, as new risk factors are found, the association of TG with a mildly reduced eGFR may need to be reassessed. Fourth, our study contained only middle-aged and elderly Chinese subjects; the present results may not be appropriate for subjects of different ages or ethnicities. Finally, the GFR based on creatinine and estimated by the CKD-EPI equation may not accurately reflect kidney function, which may have influenced the outcomes of this study. However, the gold standard method for measuring the GFR (isotope clearance measurement) is very expensive and time-consuming, so the use of creatinine-based equations to estimate the GFR is logical for large epidemiological studies. The CKD-EPI equation was more accurate and precise than the MDRD study equation and the Cockcroft-Gault equation and, therefore, may be the best choice for estimating the GFR.

In conclusion, we found that TG levels were closely associated with a mildly reduced eGFR in subjects with normal serum lipid levels. Though the evidence is not sufficient, new criteria for dyslipidemia may be needed for middle-aged and elderly Chinese subjects with a mildly reduced eGFR. Longitudinal studies are needed to explore how much the TG value should be controlled in clinical practice to have a beneficial effect on CKD.
